# Ranavirus Outbreak in North American Bullfrogs (*Rana catesbeiana*), Japan, 2008

**DOI:** 10.3201/eid1507.081636

**Published:** 2009-07

**Authors:** Yumi Une, Akiko Sakuma, Hiroki Matsueda, Katsuki Nakai, Masaru Murakami

**Affiliations:** Azabu University, Kanagawa, Japan (Y. Ume, A. Sakuma, H. Matsueda, M. Murakami); Nature Conservation Division of Shiga Prefecture, Shiga, Japan (K. Nakai)

**Keywords:** Ranavirus disease, ranavirus, Rana catesbeiana, RCV-JP virus, North American bullfrogs, amphibians, frogs, viruses, Japan, letter

**To the Editor:** Ranaviruses (family *Iridoviridae*) are emerging pathogens of farmed and wild amphibians and cause high mortality rates in these animals (*1*). These viruses are associated with massive population decreases of some species (*2*,*3*); outbreaks have been reported in the United States, Asia, Micronesia, and Europe. At the general meeting held by the International Epizootic Office in May 2008, iridoviruses of amphibians were added to the list of pathogens of wildlife that should be monitored (www.oie.int/aac/eng/Publicat/Cardsenglish/Ranavirus%20card_final.pdf, www.oie.int/eng/normes/fcode/en_chapitre_2.4.2.htm, and www.jcu.edu.au/school/phtm/PHTM/frogs/otherdiseases-viruses.htm). We report an outbreak of ranavirus disease in amphibians in Japan.

A mass die-off of wild North American bullfrog (*Rana catesbeiana*) larvae was discovered in a 1,000-m^2^ pond in western Japan. The die-off lasted from September 10 through October 20, 2008, with an epidemic peak on September 20, during which several thousand carcasses were collected daily. No dead adults of *R*. *catesbeiana* or other amphibian species were found. Fish (families Cyprinidae and Gobiidae) in the pond were unaffected.

Clinical signs in frogs were depression; lethargy; palpebral hyperemia; abdominal edema, petechiae, and erythema on the ventral surface; skin ulcers; limb and tail necrosis; and emaciation. Pathologic changes were similar in all larvae. At necropsy, subcutaneous edema, body cavity effusions, and swollen and friable livers were observed. Histologic examination showed extensive glomerular necrosis with renal tubular hyaline droplet degeneration (Appendix Figure) and various degrees of hepatic cell degeneration and necrosis. Myxosporidia were not observed within any renal tubules. Electron microscopy showed cytoplasmic ranavirus-like particles within glomerular endothelial cells. These particles were icosahedral with a diameter of ≈120 nm. Bacterial colonies were observed on the skin and within multiple organs in some larvae examined. These colonies were interpreted to be opportunistic organisms and microbial cultures were not performed.

PCR with primers M153 and M154 (*4*) amplified a ranavirus-specific gene encoding major capsid protein (MCP) from 18 bullfrog specimens. DNA sequences (584 nt, which did not include primer-annealing regions) obtained from 5 PCR products randomly selected by direct-sequencing were identical. These sequences showed highest similarities with those of *R*. *catesbeiana* virus TW07–440 (GenBank accession no. FJ207464); only 1 nt difference was observed and this difference resulted in an amino acid substitution. Amplifications with several sets of primers (M68/M69, M70/M71, M72/M73, M84/M85, and M151/M152) (*4*) and sequencing were conducted.

We determined MCP DNA sequences of 1,472 nt that included the complete coding region (nt positions 17–1408, 1,392 nt) and proximal flanking regions. Sequences were deposited in the DNA Data Bank of Japan/GenBank/European Molecular Biology Laboratory DNA databases under the accession no. AB474588. Phylogenetic analysis showed that virus detected in this study, designated RCV-JP, showed greater similarity to TW07–440 virus than to other ranaviruses, including tadpole edema virus (*5*), frog virus 3 (*6*), and *R*. *catesbeiana* virus Z (*7*). Liver tissues of fish (*Gnathopogon* spp.) that cohabitated the pond, but showed no external signs of disease, were positive for ranavirus by PCR using primers M153 and M154. Further sequence analyses are ongoing, and additional investigations of other amphibians and fishes are needed.

Live freshwater fish from several countries have been imported into Japan. However, large amounts (<1,300 tons in 2007) of live aquaculture products, including eels and other fishes, have been imported from Taiwan into Japan (www.customs.go.jp/tariff/2007_4/data/03.htm). Given that viruses that originate in Japan and Taiwan are similar, the ranavirus we detected was likely imported into Japan in an infected aquatic organism. However, an epidemiologic survey will be necessary to determine the source of the ranavirus in the pond studied. Likewise, this virus may be endemic to Japan, and a survey of native and foreign free-ranging amphibians should be conducted. Molecular analysis of ranaviruses detected in these surveys will be necessary to differentiate endemic viruses from introduced viruses.

Japan is located at middle latitudes and has a temperate climate. This country has long been geologically isolated from Asia. This isolation has resulted in the development of many diverse species of amphibians in Japan; 23 species of the order Caudata and 35 species of the order Anura. Of these species, 49 (84%) are native and 36 (62%) are listed by the Ministry of the Environment as threatened species (*8*). *R*. *catesbeiana* frogs were introduced into Japan in 1918 as a food animal, and raising them by aquaculture was widely attempted. Although they are no longer cultured, feral populations have become established throughout Japan (*9*). Ranavirus in *R*. *catesbeiana* frogs represents a serious threat to amphibians throughout Japan.

## Supplementary Material

Appendix Figure North American bullfrog (Rana catesbeiana) metamorphs infected with ranavirus RCV-JP. A) Necrosis of distal extremities (arrows) and mild abdominal swelling. Scale bar = 1 cm. B) Severe abdominal swelling caused by body cavity effusion. Scale bar = 1 cm. C) Kidney of an infected frog with necrosis of glomeruli and tubular hyaline droplet degeneration; hematoxylin and eosin stain. Scale bar = 100 m. Inset shows ranavirus-like particles; scale bar = 100 nm.
