# Supplementary figures and images for: Ranavirus Outbreak in North American Bullfrogs (Rana catesbeiana), Japan, 2008
Source: Emerg Infect Dis. 2009 Jul;15(7):1146–7. doi: 10.3201/eid1507.081636 (PMC2744262; doi:10.3201/eid1507.081636)

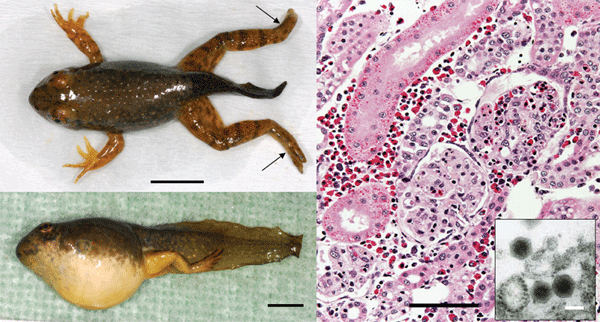

Supplement: Appendix Figure — North American bullfrog (Rana catesbeiana) metamorphs infected with ranavirus RCV-JP. A) Necrosis of distal extremities (arrows) and mild abdominal swelling. Scale bar = 1 cm. B) Severe abdominal swelling caused by body cavity effusion. Scale bar = 1 cm. C) Kidney of an infected frog with necrosis of glomeruli and tubular hyaline droplet degeneration; hematoxylin and eosin stain. Scale bar = 100 m. Inset shows ranavirus-like particles; scale bar = 100 nm. [file 08-1636_appF-s1.gif]
